# Natural Formulation Based on Diatomaceous Earth and Botanicals against Stored Product Insects

**DOI:** 10.3390/insects11090613

**Published:** 2020-09-08

**Authors:** Ivan Paponja, Vlatka Rozman, Anita Liška

**Affiliations:** 1 Faculty of Agrobiotechnical Sciences Osijek, Josip Juraj Strossmayer University of Osijek, 31000 Osijek, Croatia; paponja.ivan1@gmail.com; 2 Department for Phytomedicine, Faculty of Agrobiotechnical Sciences Osijek, Josip Juraj Strossmayer University of Osijek, 31000 Osijek, Croatia; vrozman@fazos.hr

**Keywords:** diatomaceous earth, essential oil, lavender, botanicals, stored product insects

## Abstract

**Simple Summary:**

Stored product insects play a major role in postharvest loss. In order to minimize negative effect of conventional insecticides, diatomaceous earth (DE) is one of the alternative solutions for insect control. Despite favorable effect for the environment and human health, DE has some negative side effects on the treated commodity. In order to overcome the limitations of DEs, the aim of this study was to develop natural formulation which would improve the activity of DE. Formulation (labeled as N Form) based on DE enhanced with botanicals and silica gel was tested against three major stored product insect species in wheat and barley under controlled conditions. N Form showed higher efficacy than DE, especially in barley, inducing higher mortality of all three insect species. This study provides new information about the improvement of DE effectiveness thus representing a contribution to further development of natural insecticides as a part of integrated pest management.

**Abstract:**

Diatomaceous earth (DE) has long been known as a potential protectant for stored cereals against various stored product insects. Despite favorable effect for the environment and human health, DE has some negative side effects on the treated commodity. In order to minimize negative response and to improve its efficacy, this paper represents a study of developed natural formulation based on DE SilicoSec^®^ enhanced with botanicals (essential oil lavender, corn oil, and bay leaves dust) and silica gel. The activity of formulation (labeled as N Form) was tested against *Sitophilus oryzae* (L.)*, Rhyzopertha dominica* (F.), and *Tribolium castaneum* (Herbst) in seed wheat and barley under controlled conditions. As a reference comparative value, DE SilicoSec^®^ was used. N Form showed higher efficacy than DE, especially in barley at the lowest concentration, inducing higher mortality of all three insect species. The highest average progeny inhibition was recorded in *R. dominica* population both in seed wheat and barley with 94.9% and 96.3% of inhibition, respectively, followed with *S. oryzae* and *T. castaneum* inhibition of 90.6% and 86.1%, respectively, in wheat and 94.9% and 89.7%, respectively, in barley. Results indicate that the developed natural formulation N Form enhanced the activity of DE SilicoSec^®^ using lower amount of DE dust and that it could be successfully implemented for storage of cereals as alternatives to chemical pesticides for stored product insect control.

## 1. Introduction

Stored product insects play a major role in postharvest loss, both quantitative and qualitative. Thus, it is a great challenge to minimize grain losses during storage and important to use efficient insecticides that are safe for humans and the environment.

The use of diatomaceous earth (DE) provides most of these requirements. Its main advantages are low mammalian toxicity and long persistence [1] with efficient insecticidal activity without leaving hazardous residues [2,3]. Yet, there are some limitations, which impede wider commercial use of DE, such as reduction of bulk density and flowability of grain [4,5], creation of dusty environment, and decrease in efficacy at higher grain moisture contents [6] and variable efficacy based on type of commodity (rice, sorghum, rye, corn, or wheat) [7]. Due to a desiccating mode of action, DEs are slow-acting insecticides. They are less effective against insects developing inside the kernel. Consequently, efficacy can differ among some insect species [2,5,8] and different development stages [9].

In order to overcome the limitations of DEs, diverse studies were conducted to discover new ways of DE use [10]. One attempt was mixing DE with botanicals, plant extracts [11], or essential oils (EO) [12], which revealed enhanced and synergistic effectiveness [13,14]. Silica gels, which are produced by drying aqueous solutions of sodium silicate, are very light hydrophobic powders [15]. Dehydration is also the main cause of insect death [16], but unlike DE, silica gel has the advantage of a much larger surface area than diatomaceous earth [15] and has very fast initial effectiveness [3]. 

In light of these findings, the aim was to incorporate benefits of botanicals, silica gel, and DE into a powder formulation, which would provide higher efficacy than DE used alone. In this study, the activity of developed formulation labelled as N Form was tested against three stored product insect species: the rice weevil *Sitophilus oryzae* (L.) (Coleoptera: Curculionidae), the lesser grain borer *Rhyzopertha dominica* (F.) (Coleoptera: Bostrychidae), and the red flour beetle *Tribolium castaneum* (Herbst) (Coleoptera: Tenebrionidae) under controlled conditions.

## 2. Materials and Methods

### 2.1. Natural Formulation 

Powder formulation labeled as N Form was based on diatomaceous earth (DE) SilicoSec^®^ (48% *wt*/*wt*), silica gel SIPERNAT^®^ 50 S (24% *wt*/*wt*), dried and milled bay leaves (20% *wt*/*wt*), corn oil (3% *wt*/*wt*), essential oil (EO) of lavender *Lavandula x intermedia* (2% *wt*/*wt*), and unactivated yeast as a food grade bait. All ingredients were mixed together on an electromagnetic sieve shaker (CISA RP08) using sieve with mesh size of 500 µm. Prepared N Form was kept in hermetically sealed bottles until application. 

### 2.2. Test Insects 

The insecticidal effect of the formulation N form was evaluated on three stored product insect species, two internal feeders, *S. oryzae* and *R. dominica* and one external feeder, *T. castaneum*. Test insects have been reared under the controlled conditions at 28 ± 1 °C and 65 ± 5% relative humidity (r.h.) on whole soft white wheat (for *S. oryzae* and *R. dominica*) and on mixture of wheat flour and 5% brewer’s yeast by weight (for *T. castaneum*). All adults used in the test treatments were 7–21 days old.

### 2.3. Commodity

For the experiment, Croatian varieties of wheat and barley were used. High-yielding winter barley variety Bingo (protein content 11.02% and oil content 11%) had initial moisture content (m.c.) of 15.2% and 75.8 kg hL^−1^ test weight and high-yielding winter wheat variety Anđelka (protein content 13.0% and oil content 12.5%) had 12.7% of initial m.c. and 74.7 kg hL^−1^ test weight. Moisture content and test weight of wheat and barley were measured by the GAC 2100-Agri Grain analysis computer (Dickey-john). Prior to use in bioassay, the seed was cleaned and sterilized at 50 °C and acclimated for 7 d at 28 ± 1 °C and 65 ± 5% r.h. to maintain the level of moisture content at specified limit. 

### 2.4. Bioassay in Controlled Conditions

The formulation N Form was applied as dust to seeds in 4 different concentrations (300, 400, 500, and 600 ppm). Glass jars of 200 mL volume were filled with 100 g of clean wheat or barley, respectively, and determined concentrations of the tested formulation were added. Jars were tightly closed with lids and thoroughly shaken for 30 s for equal distribution of added dust over the seeds. After the dust settled, 20 unsexed, 7–21 days old adults of *S. oryzae, R. dominica,* or *T. castaneum* were added into each jar period. Jars were closed with perforated lids and left in control conditions (28 ± 1 °C and 60 ± 5% r.h.). Adults’ mortality and progeny reduction were evaluated. Adults’ mortality was evaluated after 7 and 14 d of exposure on the same set of jars. After the assessment of adult mortality, all adults were removed and remaining seeds with laid eggs were left under controlled conditions for 7–9 weeks (for *S. oryzae* and for *R. dominica* and *T. castaneum,* respectively) to evaluate the emerged progeny. All treatments were replicated three times for each concentration, separately for each insect species and type of seed. The same procedure was followed for the untreated wheat and barley that served as control. As a reference comparative value, DE SilicoSec^®^ was used and tested at the same concentrations and within the identical treatment as N Form. 

### 2.5. Data Analysis

Generally, control mortality was, in most cases, 0%, except in barley mortality of *S. oryzae,* which ranged from 0% to 6.5%. The mortality data of exposed adult insect species (from the bioassay in controlled conditions) and progeny population (with the control included) were processed by SAS v9.3 (SAS/STAT Software 9.3 2013–2014). One-way analysis of variance of the tested variables was subjected in SAS Analyst module and the procedure of ANOVA was used. Tukey’s HSD (*p* ˂ 0.05) test was used to detect differences among means of examined traits. 

## 3. Results

### Mortality in Controlled Conditions 

In wheat, the tested formulation N Form showed high efficiency against all three insect species especially after 14 days of exposure (Table 1). The most sensitive species was *S. oryzae* with the highest mortality reached at concentration of 400 ppm, followed by *R. dominica* with 100% mortality at concentration of 600 ppm, while mortality of *T. castaneum* ranged from 76.6% to 96.6% after 14 days of exposure. Comparing the activity of N form and the activity of DE SilicoSec^®^ per each concentration, although there was no statistical difference, a higher activity of N Form was observed against *T. castaneum* and *R. dominica* after 7 and 14 days of exposure.

In barley, the tested formulation N Form induced 100% mortality of all three insect species at 600 ppm after 7 days of exposure (Table 2). There was no statistical difference among concentrations. Comparing the activity of N form and the activity of DE SilicoSec^®^, a statistical higher mortality against all three insect species was observed with N Form at lower applied concentrations (300 and 400 ppm). 

After the assessment of progeny population developed from treated adults a significant inhibition was observed at the lowest concentration of N Form in all three insect species, both in wheat and barley (Table 3). There were no statistical differences in number of adults among concentrations for each species. In barley, formulation, N Form reached higher average progeny inhibition rate of all three insect species than in wheat (94.9% and 90.6%, respectively, for *S. oryzae,* 96.4% and 95.0%, respectively, for *R. dominica,* and 89.7% and 86.1%, respectively, for *T. castaneum*). Further, N Form had higher average progeny inhibition rate than DE SilicoSec^®^, particularly in barley for all three insect species.

## 4. Discussion

This study indicates that developed formulation N Form based on DE SilicoSec^®^ enhanced with botanicals (essential oil lavender, corn oil, and bay leaves dust) and silica gel showed high levels of protection of wheat and barley against all three insect species. Among the tested species, the most sensitive in wheat and barley was *S. oryzae*, with the highest average mortality rate after 14 days postexposure (96.2% and 100.0%, respectively), followed with *R. dominica* (89.8% and 96.2%, respectively) and *T. castaneum* (84.5% and 94.9%, respectively). Generally, lower sensitivity of *Tribolium* spp. adults than *S. oryzae* and *R. dominica* to different DE formulations was proven earlier through other studies [17,18,19,20]. The basis probably lies between the combinations of physiology, morphology, genetics, and behavior response of *T. castaneum,* like differences in epicuticle, mobility through the grain mass or ability of water loss recovery [21,22,23]. Treated adults of the rusty grain beetle *Cryptolestes ferrugineus* (Stephens), *R. dominica,* and *T. castaneum* with DE (500 ppm) showed different adsorption of DE particles, which corresponds with differences in their mortality [23]. The previous author revealed that the highest number of hair-like structures, the highest density of DE particles on the body, and the highest mortality are shown by *C. ferrugineus,* compared *T. castaneum,* which has very smooth skin surface and a low level of mortality.

N Form provided complete control of *S. oryzae, R. dominica,* and *T. castaneum* in barley and of *S. oryzae* and *R. dominica* in wheat. In regard to DE SilicoSec^®^, developed formulation N Form showed higher activity against all three insect species; higher mortality rate both in wheat and barley; and higher average progeny inhibition rate, namely, in barley. Apparently, additional ingredients of formulation N Form, botanicals and silica gel, contributed to the higher efficacy of DE SilicoSec^®^, with only 48% of DE within its composition. The most important disadvantage of DE usage in stored cereals protection is the application of high concentration for successful pest control, which impairs the quality of cereals, such as physical and mechanical properties of the grain. Thus, reducing quantity of DE and at the same time, retaining and improving its efficacy can be considered as a key effect in value of the developed formulation. In a previous study [24], additive effect was also recorded, after combining inert dust originated from Croatia, botanicals, silica gel, and pyrethrin. After 6 months, formulations F1H and F2H showed higher insecticidal effect than DE Celatom^®^ Mn 51 in corn and wheat against *R. dominica.* There are many studies where DEs have been mixed with other products with the aim of improving its efficacy and reducing the limitations. Korunic and Fields [20] found that three DE-based formulations (with combinations of DE, silica gel, pyrethrin, dill essential oil, and disodium octaborate tetrahydrate—DOT and yeast) were effective at controlling insects at lower concentrations than DE alone, and at the same time, lower concentrations affected reduction of bulk density much less than DE used alone. In many cases, the synergy between DE and added substances greatly enhanced the effectiveness of a mixture and therefore, the needed effective concentrations of DE had been greatly reduced by approximately 4 to 10 times in the comparison with concentrations of DE when used alone [25]. In this study, N Form achieved higher average progeny inhibition rate than DE SilicoSec^®^, particularly expressed in barley for all three insect species. It could be explained with the faster activity of N Form than activity of DE SilicoSec^®^. The mortality rate of treated parents was higher after 7 days postexposure, which was directly reflected in lower progeny production. Probably, faster activity is related to additional products within the formulation N Form. Namely, silica gel Sipernat^®^ 50 S could have contributed to the fast initial effectiveness [3] and EO of lavender possessing the multiple modes of action against insects [26]. Stronger and faster mortality induced by formulation N Form consequently led to higher inhibition of progeny population compared to DE SilicoSec^®^ used alone.

Overall, formulation N Form showed higher efficacy against tested insect species in barley than in wheat. Unlike in wheat, 100% mortality of all three insect species was reached only after 7 days postexposure. Consequently, the progeny inhibition rate of all three insect species was higher in barley. The explanation for those differences can be seen through multiple perspectives. First of all, DEs and formulations based on DE are not equally effective on all grain types [27], which is related to different adherence of DE influenced by different kernel size and protein content [28] or oil content. The effect on different types of commodities like rice, sorghum, rye, corn, and wheat was investigated on the efficacy of DE [7,29], based on physiochemical characteristics of the grain as a relevant factor in treatment effectiveness. In addition, a significant difference in efficacy of DE was observed in different classes or varieties of grain [30]. Second, different physiology and morphology of kernels, such as kernel hardness and nutritional value, can attribute to development and reproduction rate of insect species [28,31]. Contrary to N Form, the activity of DE SilicoSec^®^ was lower in barley than in wheat against *S. oryzae* and *R. dominica,* which is another proof of enhanced effectiveness of the developed formulation. 

## 5. Conclusions

Overall, results of this study indicate that natural formulation N Form, based on botanicals, silica gel, and DE, was highly effective against three major stored product insect species *S. oryzae, R. dominica,* and *T. castaneum* in wheat and barley. Achieving stronger efficacy than DE SilicoSec^®^ applied alone, this study provides new information about the improvement of DE effectiveness, thus representing a contribution to further development of natural insecticides as a part of integrated pest management program. Future trials will be designed to test the activity of N Form against other stored insect species, on different types of surfaces in order to examine the possibility of use as a surface treatment of empty storage facilities. Additionally, tests of influence of N Form parameters of grain quality are planned to be implemented.

## Figures and Tables

**Table 1:** Mean (% ± SD) mortality of *Tribolium castaneum, Sitophilus oryzae,* and *Rhyzopertha dominica* adults after 7 and 14 d of exposure to treated wheat seeds with formulation N Form and DE SilicoSec^®^.

Treatment	Concentration (ppm)	Mean (% ± SD ^a^) Mortality	
		7 d of Exposure	14 d of Exposure
*Tribolium castaneum*			
**N Form**	300	23.3 ± 15.27	76.6 ± 12.58 ab
	400	45.0 ± 13.22	86.6 ± 14.53 a
	500	40.0 ± 35.00	78.3 ± 29.29 ab
	600	30.0 ± 13.22	96.6 ± 5.77 a
**SilicoSec^®^**	300	3.3 ± 2.88	36.6 ± 5.77 b
	400	16.6 ± 2.89	55.0 ± 5.0 ab
	500	21.6 ± 12.58	70.0 ± 13.22 ab
	600	48.3 ± 24.66	88.3 ± 20.20 a
	F/P	2.18/0.0934	4.83/0.0044
*Sitophilus oryzae*			
**N Form**	300	71.6 ± 10.40 b	86.6 ± 18.92
	400	95.0 ± 5.00 a	100.0 ± 0.00
	500	98.3 ± 2.88 a	100.0 ± 0.00
	600	98.3 ± 2.88 a	98.3 ± 2.88
**SilicoSec^®^**	300	86.6 ± 7.63 ab	98.3 ± 2.88
	400	81.6 ± 7.63 ab	100.0 ± 0.00
	500	86.6 ± 7.63 ab	100.0 ± 0.00
	600	96.6 ± 5.77 a	100.0 ± 0.00
	F/P	6.06/0.0014	1.36/0.2875
*Rhyzopertha dominica*			
**N Form**	300	63.3 ± 15.27 ab	76.6 ± 20.20 ab
	400	66.6 ± 15.27 ab	85.0 ± 18.02 ab
	500	86.6 ± 15.27 a	98.3 ± 2.88 a
	600	83.3 ± 5.77 a	100.0 ± 0.00 a
**SilicoSec** ^®^	300	36.6 ± 7.63 b	63.3 ± 7.63 b
	400	61.6 ± 10.40 ab	85.0 ± 13.22 ab
	500	80.0 ± 10.00 a	90.0 ± 10.00 ab
	600	71.6 ± 17.55 ab	90.0 ± 5.0 ab
	F/P	4.67/0.0051	3.06/0.0301

^a^ Means in the same column within each insect species followed by the same letters are not significantly different (for all treatments *df* = 7, 23; Tukey’s HSD, *p* = 0.05).

**Table 2:** Mean (% ± SD) mortality of *Tribolium castaneum, Sitophilus oryzae,* and *Rhyzopertha dominica* adults after 7 and 14 d of exposure to treated barley seeds with formulation N Form and DE SilicoSec^®^.

Treatment	Concentration (ppm)	Mean (% ± SD ^a^) Mortality	
		7 d of Exposure	14 d of Exposure
*Tribolium castaneum*			
**N Form**	300	73.3 ± 24.66 ab	81.6 ± 14.43 a
	400	96.6 ± 5.77 a	98.3 ± 2.88 a
	500	96.6 ± 2.88 a	100.0 ± 0.00 a
	600	100.0 ± 0.00 a	100.0 ± 0.00 a
**SilicoSec^®^**	300	10.0 ± 8.66 c	50.0 ± 18.02 b
	400	46.6 ± 23.9 bc	90.0 ± 10.00 a
	500	80.0 ± 8.66 ab	96.6 ± 5.77 a
	600	83.3 ± 7.63 ab	100.0 ± 0.00 a
	F/P	16.42/˂0.0001	10.57/˂0.0001
*Sitophilus oryzae*			
**N Form**	300	95.0 ± 8.66 a	100.0 ± 0.00
	400	95.0 ± 5.00 a	100.0 ± 0.00
	500	98.3 ± 2.88 a	100.0 ± 0.00
	600	100.0 ± 0.00 a	100.0 ± 0.00
**SilicoSec^®^**	300	63.3 ± 10.40 b	98.3 ± 2.88
	400	75.0 ± 10.00 b	91.6 ± 14.43
	500	96.6 ± 5.77 a	100.0 ± 0.00
	600	100.0 ± 0.00 a	100.0 ± 0.00
	F/P	12.72/˂0.0001	0.95/0.5005
*Rhyzopertha dominica*			
**N Form**	300	56.6 ± 27.33 abcd	86.6 ± 7.63 bc
	400	78.3 ± 7.63 abc	98.3 ± 2.88 ab
	500	91.6 ± 7.63 ab	100.0 ± 0.00 a
	600	100.0 ± 0.00 a	100.0 ± 0.00 a
**SilicoSec^®^**	300	36.6 ± 5.77 d	68.3 ± 2.88 d
	400	40.0 ± 8.66 cd	71.6 ± 2.88 d
	500	63.3 ± 20.81 abcd	85.0 ± 8.66 c
	600	90.0 ± 10.00 ab	98.3 ± 2.89 ab
	F/P	9.20/0.0001	23.94/˂0.0001

^a^ Means in the same column within each insect species followed by the same letters are not significantly different (for all treatments *df* = 7, 23; Tukey’s HSD, *p* = 0.05).

**Table 3:** Progeny (F1) population of three tested insect species after parent exposure to wheat and barley seeds treated with formulation N Form and DE SilicoSec^®^.

Treatment	Concentration (ppm)	*S. Oryzae*		*R. Dominica*		*T. Castaneum*	
		Number of Adults Mean ± SD ^a^	Inhibition (%)	Number of Adults Mean ± SD ^a^	Inhibition (%)	Number of Adults Mean ± SD ^a^	Inhibition (%)
Wheat							
**N Form**	0	372.6 ± 155.93 a	–	73.3 ± 31.56 a	–	11.33 ± 5.03 a	–
	300	103.0 ± 65.50 b	72.4	4.6 ± 0.57 b	93.7	3.3 ± 1.15 b	70.8
	400	19.6 ± 13.01 b	94.7	4.0 ± 1.73 b	94.5	2.0 ± 1.73 b	82.3
	500	10.3 ± 4.93 b	97.2	4.3 ± 3.05 b	94.1	1.0 ± 1.00 b	91.2
	600	7.3 ± 3.78 b	98.0	2.0 ± 1.00 b	97.3	0.0 ± 0.00 b	100.0
	F/*P*	12.68/0.0006		14.40/0.0004		10.04/0.0016	
**SilicoSec^®^**	0	372.6 ± 155.93 a	–	73.3 ± 31.56 a	–	11.33 ± 5.03 a	–
	300	40.3 ± 15.69 b	89.2	14.6 ± 3.51 b	80.1	1.0 ± 0.00 b	91.2
	400	32.6 ± 25.71 b	91.3	8.3 ± 8.50 b	88.7	1.3 ± 0.57 b	88.5
	500	33.6 ± 10.40 b	91.0	3.3 ± 0.57 b	95.5	0.0 ± 0.00 b	100.0
	600	20.3 ± 3.51 b	94.6	4.0 ± 4.35 b	94.5	0.6 ± 1.15 b	94.7
	F/*P*	13.79/0.0004		12.06/0.0008		12.58/0.0006	
Barley							
**N Form**	0	265.6 ± 60.18 a	–	355.3 ± 103.01 a	–	20.6 ± 5.5 a	–
	300	19.6 ± 11.93 b	92.6	31.3 ± 12.58 b	91.2	6.6 ± 1.52 b	68.0
	400	10.3 ± 12.09 b	96.1	8.3 ± 5.85 b	96.7	1.3 ± 1.15 b	93.7
	500	13.6 ± 7.63 b	94.9	7.6 ± 1.52 b	97.9	0.3 ± 0.57 b	98.5
	600	10.6 ± 8.08 b	96.0	1.3 ± 1.52	99.6	0.3 ± 0.57 b	98.5
	F/*P*	47.30/˂0.0001		32.86/˂0.0001		32.61/˂0.0001	
**SilicoSec^®^**	0	265.6 ± 60.18 a	–	355.3 ± 103.01 a	–	20.6 ± 5.5 a	–
	300	45.3 ± 14.01 b	82.9	51.3 ± 16.07 b	85.6	6.0 ± 1.73 b	70.9
	400	32.3 ± 13.20 b	87.8	31.3 ± 4.16 b	91.2	3.0 ± 2.64 b	85.4
	500	27.3 ± 16.25 b	89.7	31.6 ± 4.50 b	91.1	1.3 ± 1.52 b	93.7
	600	19.6 ± 6.65 b	92.6	11.6 ± 3.78 b	96.7	1.6 ± 1.52 b	92.2
	F/*P*	38.65/˂0.0001		29.07/˂0.0001		21.94/˂0.0001	

^a^ Means in the same column within each treatment followed by the same letters are not significantly different (in all cases *df* = 4, 14; Tukey’s HSD, *p* = 0.05).
